# The CCCTC-Binding Factor (CTCF) of *Drosophila* Contributes to the Regulation of the Ribosomal DNA and Nucleolar Stability

**DOI:** 10.1371/journal.pone.0016401

**Published:** 2011-01-20

**Authors:** Paola A. Guerrero, Keith A. Maggert

**Affiliations:** 1 Department of Biochemistry/Biophysics, Texas A&M University, College Station, Texas, United States of America; 2 Department of Biology, Texas A&M University, College Station, Texas, United States of America; Duke University, United States of America

## Abstract

In the repeat array of ribosomal DNA (*rDNA*), only about half of the genes are actively transcribed while the others are silenced. In arthropods, transposable elements interrupt a subset of genes, often inactivating transcription of those genes. Little is known about the establishment or separation of juxtaposed active and inactive chromatin domains, or preferential inactivation of transposable element interrupted genes, despite identity in promoter sequences. CTCF is a sequence-specific DNA binding protein which is thought to act as a transcriptional repressor, block enhancer-promoter communication, and delimit juxtaposed domains of active and inactive chromatin; one or more of these activities might contribute to the regulation of this repeated gene cluster. In support of this hypothesis, we show that the *Drosophila* nucleolus contains CTCF, which is bound to transposable element sequences within the *rDNA*. Reduction in CTCF gene activity results in nucleolar fragmentation and reduced *rDNA* silencing, as does disruption of poly-ADP-ribosylation thought to be necessary for CTCF nucleolar localization. Our data establish a role for CTCF as a component necessary for proper control of transposable element-laden *rDNA* transcription and nucleolar stability.

## Introduction

Electron micrographs of transcribing *rDNA* loci by O. L. Miller, Jr. and colleagues have provided a cytological foundation to subsequent studies showing histone modification-, DNA methylation-, and regulatory RNA-mediated epigenetic regulation of the *rDNA* loci across kingdoms [1], [2]. Many studies have led to the prevailing view that only about one-half of the *rDNA* cistrons are active, while the remainder are kept silent through epigenetic modification of chromatin structure. Although much is now known about *rDNA* chromatin structure, relatively little is known about the decisions of how many and which cistrons are inactive, whether all cell types make this decision, and once made how active and inactive chromatin domains are kept separate. Balance between activating and repressive factors may control the ratio [3], although such a model does not account for the preferential inactivation of the subset of cistrons that might be interrupted by transposable element. To account for this, a simple model suggests a sequence-specific repressor might inactivate some *rDNA* cistrons, and a boundary element may maintain separation of active and inactive regions [1], [4]. Although RNA Polymerase III, RNA Polymerase I regulators, or DNA-replication proteins may serve to separate domains in yeasts [5], [6], [7], [8], little is known of how similar regulation may be accomplished in animals and plants.

In arthropods, the R1 and R2 non-long-terminal-repeat (non-LTR) retrotransposable elements interrupt a high proportion of *35S rDNA* cistrons (17%–67% *rDNA* copies are interrupted by R1, 2%–28% by R2, and up to 16% by both) [9], and molecular and cytological evidence show that these are almost always inactivated [10], [11]. These elements are inserted in a conserved site within the *28S* subunit and are colinearly transcribed with the *35S rDNA* [12], [13], showing that transcriptional silencing due to their presence affects the *rDNA* promoter approximately four kilobases away.

CTCF is a protein with complex roles in gene regulation, having been shown to act as both transcriptional activator and repressor, and be responsible for two features of genomic “boundary elements,” namely the abilities to separate chromatin with activating and inactivating histone modifications and to block enhancer-promoter interactions (recently reviewed in [14]). CTCF plays regulatory roles in the large Homeotic gene complexes of flies and mammals [15], [16], is thought to be necessary to maintain monoallelic expression of genomic imprinted loci in mouse and humans [17], and binds the inactive (dosage compensated) female mammalian X chromosome [18]. Hence, it possess the properties expected for a protein that might regulate and separate interspersed active and inactive *rDNA* cistrons. Unraveling the overlapping and separate properties of CTCF has been difficult, since consensus DNA binding sites, interaction partners, and genetic properties have proven difficult to exhaustively enumerate [15], [19].

Torrano and colleagues noted that CTCF moves to the nucleoli of terminally-differentiated mammalian (human and rat) cells [20]. It has been suggested that the localization might be a necessary step for CTCF to regulate the euchromatin [21], implying that it has no active role in the nucleolus. This view is perhaps appealing because of the example of p53 and ARF, whose regulation includes facultative nucleolar retention as means of gene product regulation [22], [23]. However, Torrano and colleagues showed over-expression of CTCF resulted in reduced nascent nucleolar transcription and argued for a direct role in transcriptional regulation, and recently van de Nobelen and colleagues showed CTCF at the *rDNA* promoter [24].

We directly addressed whether CTCF is also found in the nucleolus of *Drosophila*, binds to the *rDNA*, regulates its expression, and influences the stability of the nucleolus. In the course of our work, we discovered that CTCF is not solely a marker for terminal differentiation since it is nucleolar in many non-differentiated cell types. We found that CTCF binds to at least one specific site within the transposable elements of the repeated *rDNA* cistron, which contributes to a model for regional regulation of *rDNA* expression. We used RNAi-mediated reduction of gene activity, mutation of the gene in whole animals, and disruption of the poly-ADP-ribosylation pathway that modifies CTCF to directly determine if endogenous CTCF is necessary for normal *rDNA* silencing, and showed that all treatments resulted in both increased *rDNA* expression, expression of *rDNA*-associated transposable elements and transgenic marker gene expression, and increased nucleolar instability.

## Results

**Figure 1:**
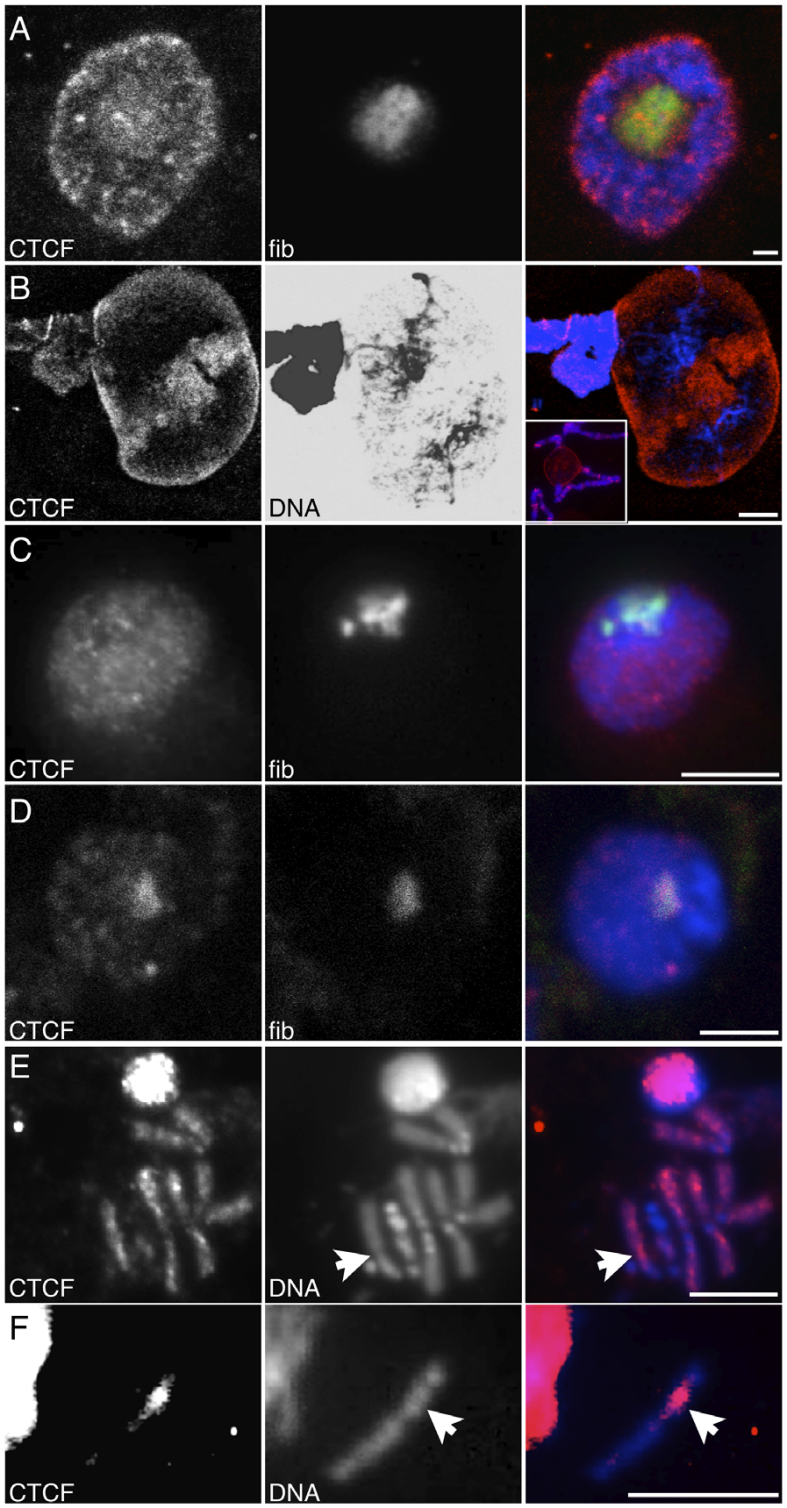
Indirect immunofluorescence reveals CTCF as a component of the nucleoli of *Drosophila* cells. (A) Confocal microscopy of whole mount third instar larval salivary gland nucleus. CTCF and fibrillarin (fib) shown separately, and merged with DNA (blue). (B) Higher magnification of nucleolus showing CTCF, DNA, and color merge. The DNA-only separation has been inverted and non-linearly adjusted for bright and contrast to reveal the filamentous structure of the DNA within the nucleolus. Inset in merged image shows a different nucleus with the CTCF-containing nucleolus in the context of CTCF-banded chromosome arms. (C) Epifluorescence microscopy of S2 tissue culture cell nucleus. CTCF and fibrillarin shown separately, and merged with DNA. (D) Confocal microscopy of third instar larval diploid interphase neuroblast nucleus. CTCF and fibrillarin shown separately, and merged with DNA (blue). (E) Epifluorescence microscopy of condensed mitotic *X* chromosome (arrow points to *rDNA* locus) from a third instar larval diploid metaphase neuroblast. The panoply of CTCF sites in the euchromatin are visible as immunofluorescence on the chromosome arms. (F) Epifluorescence microscopy of condensed mitotic *Y* chromosome from a third instar larval metaphase diploid neuroblast. For (E) and (F), CTCF is shown separately from a color merge with DNA. Scale bars 5 µm.

To determine if CTCF plays a role in *rDNA* regulation in *Drosophila*, we first had to ascertain whether it could be detected in nucleoli of numerous different cell types, and moreover to determine if it is generally used to regulate *rDNA*. We observed strong immunofluorescence signal for CTCF in the nucleoli of differentiated salivary glands of third instar *Drosophila* larvae, showing that it is cytologically associated with the *rDNA*, and supporting our belief that the biology of *Drosophila* CTCF may be similar to that of mammals. Even in occasional nuclei with multiple nucleoli, CTCF was found to overlap with all focal localization of fibrillarin, a marker for the fibrillary component of the nucleolus. Nucleolar localization of CTCF was in addition to a focal nucleoplasmic staining (Figure 1A) [16], [21], [25], [26]. At higher magnification, we observed that CTCF did not conform to any obvious landmarks of DNA within the nucleolus, although it was largely excluded from the visible DNA threads and foci (Figure 1B). Unlike mammalian tissue culture and nervous tissue, nucleolar CTCF did not require terminal differentiation and cessation of division in *Drosophila* since we observed CTCF in the nucleoli of undifferentiated cycling interphase S2 tissue culture and larval neuroblast cells (Figures 1C, D). The amount of nucleolar CTCF differed in those cell types, in the former it was neither enriched nor excluded but appeared similar to levels in the non-nucleolar chromatin, while in the latter it was moderately enriched over the amount found in the chromatin. Many non-nucleolar nuclear proteins are seen to be excluded from the nucleolus, and so the lack of CTCF exclusion is indicative of some localization even if it is not enriched in this compartment; this is especially true given the thousands of euchromatic binding sites to which it is being compared [27].

Nonetheless, we wished to observe clear CTCF nucleolar localization, and so used a stage of the cell cycle when binding to nucleolar DNA is cytologically distinct. We therefore detected CTCF in the secondary constrictions (locations of the nucleolar organizing ribosomal DNA) on neuroblast sex chromosomes (Figure 1E, F). *rDNA* localization is the only heterochromatic binding that we could detect, however the strong signal from the euchromatic compartments of the genome limited our ability to detect CTCF in distal heterochromatic blocks that juxtapose euchromatin. This localization at a time when nucleoli are disassembled and transcriptionally silent suggests localization is not due solely to protein-protein or protein-rRNA interactions in a mature nucleolus, but instead is due to direct DNA binding by CTCF.

**Figure 2:**
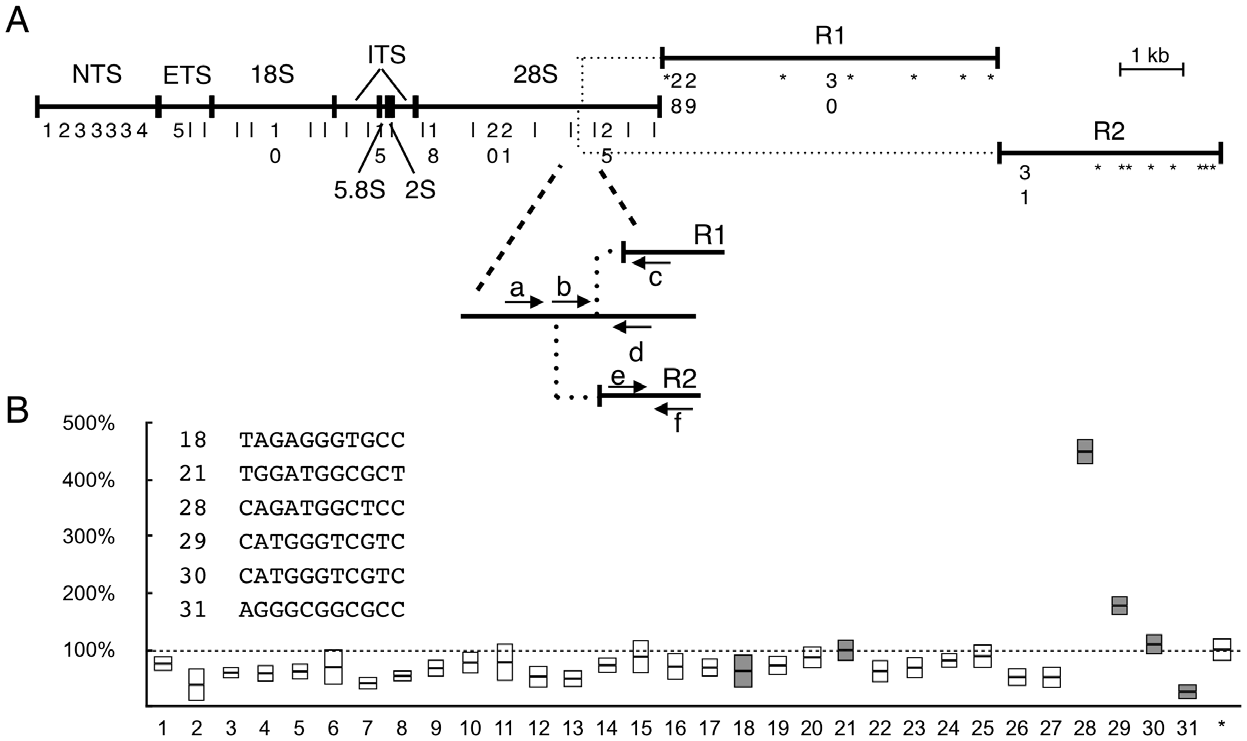
Chromatin Immunoprecipitation of CTCF identifies binding to the *rDNA* locus. (A) Map showing structure of typical *rDNA* repeat unit. NTS  =  non-transcribed spacer, ETS  =  external transcribed spacer, ITS  =  internal transcribed spacers, *18S*, *5.8S*, *2S*, and *28S* are final rRNA products, R1 and R2 are transposable element insertions (dotted lines indicate insertion sites in the 28S). Numbers indicate location of potential or predicted CTCF binding sites - all sites are shown, indicated either by numbers or by vertical hash marks. Asterisks indicate location of near-consensus sites within R1 and R2, collectively used as an “out-group.” Blow-out shows detail around R1 and R2 insertion sites in the *28S* sequence; primers “a”–“f” are used for R1-, R2-, and uninserted *35S* specific transcript detection. (B) Real-Time PCR quantification of amplification using DNA purified from chromatin immunoprecipitated by anti-CTCF antibodies. Data are presented as average boxed by pooled standard deviations of triplicate samples from three independent experiments. White data are from sites that do not match *Drosophila* CTCF consensus, gray data (18, 21, 28–31) match the *Drosophila* consensus. All data are normalized to the pooled average of the outgroup data (*), which was then defined as 100% (dashed line). Sequences show CTCF consensus sites (18, 21, 28–31).

CTCF is a sequence-specific Zinc-Finger DNA binding protein [28], and we thought it was likely to bind the *rDNA* directly. To confirm this, we predicted potential CTCF binding sites in the entire *rDNA* sequence including the non-transcribed spacer (NTS) that separates the *35S* primary transcription units, and the *28S*-interrupting R1 and R2 arthropod transposable elements [29], using the Patser algorithm informed by two different published *Drosophila* CTCF consensus sequences (Figure 2A) [15], [19], [30]. We identified six potential sites (sites 18, 21, 28–31, data in gray), and manually scanned the R1 and R2 sequences for similar potential consensus sites that differed in only one nucleotide of the core conserved consensus, which identified an additional 15 sites which served as an expected “negative” out-group (asterisks). In addition to potential binding sites identified by Patser, we designed primers to amplify sequences approximately every 350 base pairs to test potential non-consensus binding across the entire *35S rDNA* transcription unit (sites 5–17, 19–20, 22–27). We did not identify any sequences in the NTS that were similar to the CTCF consensus, although sites have been reported in the human *rDNA* NTS [24], [31], so we designed primers for chromatin immunoprecipitation of the NTS (sites 1–4) regardless of lack of obvious consensus. Of the 46 tested sites, only one showed robust binding using chromatin immunoprecipitation with antibodies raised against CTCF (Figure 2B, site 28). This site was in the DNA that corresponds to the R1 element, near the beginning of this transposable element. Although one other site (site 29) showed moderate but statistically significant immunoprecipitation, its close linkage to site 29 makes ancillary immunoprecipitation from incomplete DNA shearing a likely explanation. Even for site 28, the relative enrichment by chromatin immunoprecipitation was modest relative to the positive control of the *Fab-8* element (which had approximately 40-fold enrichment over background, data not shown) [15], but the biology of the *rDNA* locus makes chromatin immunoprecipitation potentially insensitive to occupancy at this locus, since we must consider that of the hundreds of copies of the cistron, only a fraction are silent and thus possess corresponding histone modifications or regulatory protein binding. For these reasons, we expect our chromatin immunoprecipitation signals to be lower than expected occupancy at any subset of *rDNA* cistrons.

The location of CTCF binding relative to R1 is consistent with *CTCF*-mediated silencing of inserted *35S* cistrons, either by direct repression or by separating active (expressed) from inactive (“heterochromatic”) compartments with R1-inserted cistrons as boundaries. A recent model proposed by Eickbush and colleagues is based on data suggesting proximity to R1-inserted elements is causal in *rDNA* cistron silencing, and this corresponds to cytological evidence of silenced inserted rRNA genes [10], [32]. Based on this hypothesis, and prior reports showing that CTCF may act as a transcriptional repressor [20], [28], we predicted that CTCF acts as a direct transcriptional repressor of nearby *rDNA*.

The ribosomal RNAs are the most abundant RNA species in the cell, and have very long half-life once assembled into ribosomes [33]. Our experiments are able to detect small changes in rRNA expression by measuring steady-state rRNA levels [34], however the pool of stable rRNAs is vast, and so changes due to alteration of transcriptional activity was expected to be small or statistically indistinguishable. Thus we additionally chose to assess expression of R1 and R2 and abundance of unprocessed rRNA junctions to monitor *rDNA* expression. In order to test the hypothesis that CTCF represses *rDNA* transcripts, we reduced *CTCF* gene activity in S2 cells using double-stranded RNA treatment directed at CTCF. Efficacy of treatment was assessed using immunofluorescence and Reverse Transcriptase Real-Time PCR, and transcriptional output of the *rDNA* was measured using Reverse Transcriptase Real-Time PCR of the *35S*, R1 and R2 RNAs (Figure 2A; primers b-c for expression of R1-inserted rRNA, e-f for R2, and a-d for uninterrupted *rDNA*). We used untreated cells and cells treated with RNAi directed at *LacZ* as parallel control, and measurement of *Rho1* as internal normalizing control.

**Figure 3:**
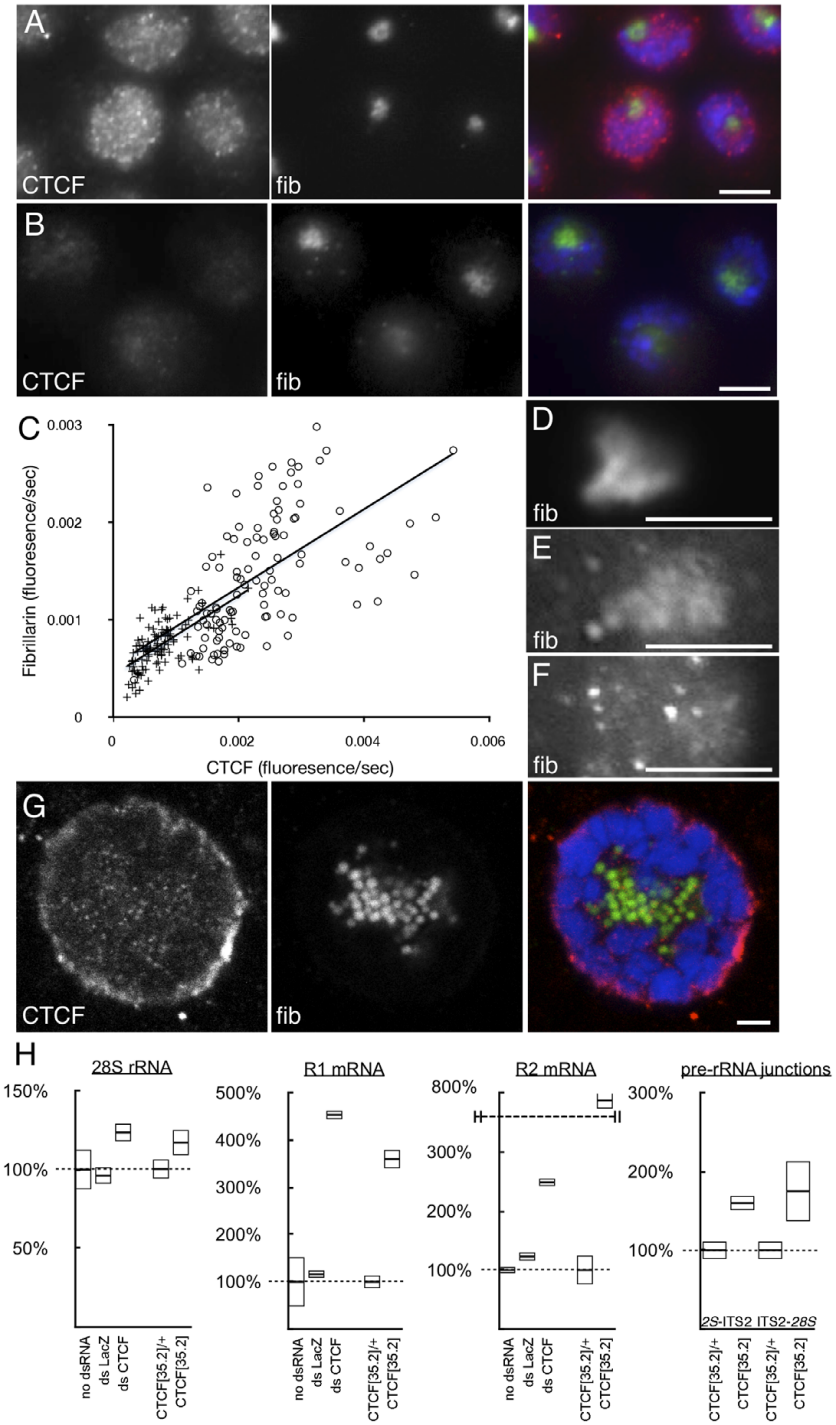
RNAi-mediated or mutational reduction of CTCF gene activity disrupts nucleolar structure and increases *rDNA* expression. (A) Indirect immunofluorescence detection of CTCF in S2 cell culture nuclei. (B) CTCF immunodetection after three-day treatment of double-stranded RNA directed at *CTCF*. Images from (A) and (B) are presented with the same exposure/bright/contrast conditions. (C) Quantification of all data from untreated (circles) and double-stranded RNA treated (crosses) cells. X-axis shows CTCF intensity (fluorescence per unit time, corrected to DNA), y-axis shows fibrillarin intensity, and regression lines are for separate datasets. (D) Higher magnification of fibrillarin-containing nucleolus from control cell treated with double-stranded RNA directed at *LacZ*. (E) Higher magnification of fibrillarin-containing nucleolus from cell treated with double-stranded RNA directed at CTCF. (F) As in (E), but a more pronounced nucleolar vesiculation/disruption phenotype. (G) Salivary gland nuclei derived from third instar larvae mutant for *CTCF*. In images from (A), (B), and (G), CTCF and fibrillarin (fib) are shown separately, and merged with DNA (blue). (H) In S2 cell culture, no double-stranded RNA treatment, or treatment with double-stranded RNAs directed at *LacZ* have no effect on *28S* rRNA (Figure 2, primers a–d), R1 (Figure 2, primers b–c) or R2 (Figure 2, primers e-f) mRNA levels, or pre-rRNA unprocessed junctions (*2S*-ITS2 and ITS2-*28S*) but treatment with double-stranded RNAs directed at *CTCF* increases *28S*, R1, R2, and pre-RNA junction RNA species. Whole intact animals bearing homozygous mutation of *CTCF* ^35.2^ results in increased *28S*, R1, R2, and pre-RNA junction RNA species compared to heterozygous *CTCF* ^35.2^/+ controls. Note discontinuity in ordinate for final R2 datum (end-hashed dashed line). All data are normalized (100%, dashed lines) to untreated cells (“no dsRNA”) and heterozygous animals (“CTCT/+”). Scale bars 5 µm.

**Figure 4:**
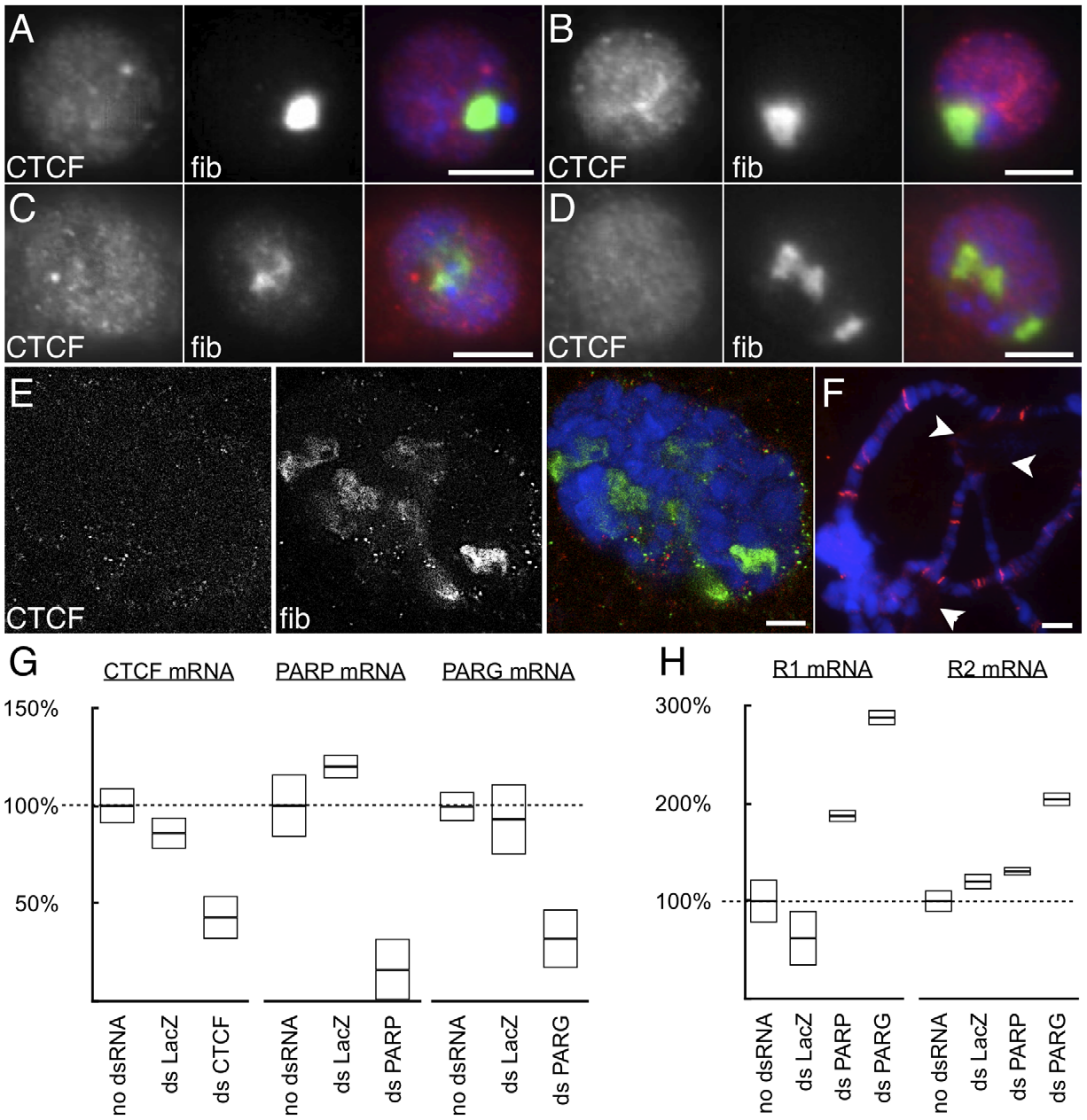
Disruption of poly-ADP-ribosylation decreases nucleolar CTCF, disrupts nucleolar structure, and increases *rDNA* expression. (A) Indirect immunofluorescence detection of CTCF and fibrillarin (fib) in S2 cell culture, and (B) after treatment with double stranded RNA directed at *LacZ*. (C) Structure of nucleoli after treatment with control double-stranded RNA directed at Poly-ADP-Ribose Polymerase (PARP), or (D) Poly-ADP-Ribose Glycohydrolase (PARG). (E) Confocal microscopy of whole mount third instar larval salivary gland nuclei derived from PARG mutants. In all preceding images, CTCF and fibrillarin (fib) are shown separately, and merged with DNA (blue). (F) Squashed chromosomes from whole mount third instar larval salivary gland nuclei show retention of CTCF at euchromatic bands but loss from the nucleolus (arrowheads). (G) Double-stranded RNA directed at CTCF, PARP, or PARG and their effects on mRNA level. (H) R1 and R2 mRNA expression after treatment with double-stranded RNAs directed at LacZ (control), PARP, and PARG. For (G) and (H), data are normalized to untreated cells (100%, dashed lines). Scale bars 5 µm.

Three day double-stranded RNA treatment resulted in a large fraction of cells with reduction of CTCF detectable by immunofluorescence (Figure 3A–F). Prior to treatment, CTCF was found in the nucleolus and foci throughout the nucleus. After treatment, CTCF was overall reduced, undetectable in many cells, and absent from foci. Integration of fluorescence showed that treatment reduced CTCF to 31.7% (±19.1% S.D.) of wild-type levels. What little CTCF remained appeared equally reduced in both the chromatin and the nucleolus, which argues against CTCF recruitment to the nucleolus as a means of sequestration of excess protein. Cells with reduced CTCF exhibited clear and reproducible disruption to the fibrillary component of the nucleoli as fibrillarin immunolocalization was either reduced or appeared more diffuse and fragmented (Figure 3B, D, E, F), in severe cases vesiculating into small foci. Fluorescence intensity co-varied with CTCF fluorescence (Figure 3C) with regression R^2^ = 0.32 in wild-type and 0.39 in cells with RNA-mediated CTCF reduction. This disruption shows that CTCF is necessary for the proper structure of the nucleolus, a common feature of regulators of *rDNA* expression (*e.g.*, chromatin modifying enzymes, RNA Polymerase I) [20], [34], [35], [36], [37]. Such disruption was not observed when cells were treated with double-stranded RNA directed at *LacZ*, *GFP*, or *Rho1*, or in untreated cells. In the population of S2 cells with RNAi-mediated reduction of CTCF (Figure 4G), we observed increased R1 and R2 expression after three days (Figure 3H), in contrast to untreated cells or cells treated with double-stranded RNA directed at *lacZ*. Expression of R1-inserted *rDNA* increased 4.5-fold, while R2 increased 2.5-fold. Although we did not detect any binding of CTCF to the R2 element sequence, the R1 and R2 inserts are thought to cluster in the repeat arrays, and so may share co-regulation [11]. Additionally, we detected an increase in steady-state levels of the uninterrupted *28S* rRNA; although significant, the increase was small possibly due to the already considerable pool of stable (ribosome-bound) rRNAs.

It is possible to address CTCF function in intact animals using mutations that eliminate detectable CTCF protein. We used a previously characterized hypomorph, CTCF^35.2^ [16]. Mutants are lethal as pharate adults, the late stage presumably owing to perdurance or maternal mRNA and protein [16]. We could therefore generate and analyze third instar larvae with reduced CTCF activity. In salivary glands obtained from *CTCF* ^35.2^ homozygotes, we again observed dramatic vesiculation of nucleolar structure (Figure 3G), similar to the disruption we observed in S2 cell culture. These nucleoli were also reminiscent of those seen upon mutation of chromatin modifying proteins known to regulate the *rDNA*, or upon reduction of the *rDNA* arrays [34], [35]. In both of those cases, copies of the rRNA genes were lost, resulting in a decrease in array size. To determine if the same *rDNA* array size reduction was an effect of CTCF mutation, we outcrossed flies bearing CTCF alleles derived from two independent sources to females of genotype *C*(*1*)*DX*, *y f bb* ^0^/*Y,B* ^S^. Female offspring have a paternal *Y* chromosome linked *rDNA* array as the sole source of *rDNA* and nucleolar organizer since the *C*(*1*)*DX* compound-*X* chromosome is entirely devoid of the *rDNA* [34], [38], [39]. All female offspring exhibited a strong bobbed phenotype, a hallmark of reduced *rDNA*. The phenotype was identical in penetrance and expressivity in both *CTCF*/+ and +/+ offspring, indicating the bobbed phenotype mapped to the *Y* chromosome common to all females, the location of the *rDNA*. We measured *rDNA* copy number using Real Time Polymerase Chain Reaction and discovered them to be 62.5% (±3.2% S.E.M.) the size of the *wild-type* controls. Despite the decrease in *rDNA* copy number, CTCF mutant animals dissected from pupal cases also showed increases in R1 and R2 expression, and *35S* rRNA expression relative to heterozygous sibling animals (Figure 3H, *CTCF* ^35.2^/+ *vs. CTCF* ^35.2^), similar to the increased expression of the *rDNA* we observed in S2 cells when CTCF mRNA and protein levels were reduced. We additionally detected increased abundance of unprocessed junctions (*2S*-ITS2 and ITS2-*28S*), which normally are efficiently processed and do not appear in the stable ribosome-bound pool of rRNA, indicating an increase in nascent transcription due to reduced CTCF activity.

In mammalian cells, both DNA binding and CTCF localization in the nucleolus require an active poly-ADP-ribosylation/glycosylation cycle [40], and mammalian nucleolar structure is affected by 3-aminobenzamide treatment [20] which inhibits this cycle. In *Drosophila* mutated for either enzyme responsible for the cycle (PARP or PARG), the nucleolus is seen to fragment [41], [42], [43] similar to what we describe for mutants of CTCF. Mutation of either component is expected to result in a similar phenotype, since PARP is itself poly-ADP-ribosylated, which leads to its inhibition. PARP requires PARG to be reactivated, and hence reduction of either gene product results in an inhibition of a robust poly-ADP-ribosylation cycle and a net decrease in this post-translational modification. Therefore, we reasoned that the disruption of nucleoli in PARP or PARG mutants may be a consequence of reduced nucleolar CTCF. We predicted that disrupting PARP and PARG would not only alter the structure of the nucleolus, but would (1) reduce CTCF in the nucleolus, and (2) reduce silencing of the *rDNA*. We tested these predictions by reducing PARP and PARG activities using double-stranded RNA treatment of S2 cells. Our results showed that reduction of PARP or PARG resulted in nucleolar disruption similar to that seen in cells with reduced CTCF (Figure 4A–D), although to a lesser degree. Genetic mutations of PARG resulted in disrupted localization of CTCF, including a loss from the nucleolus (Figure 4E–F). We did not detect obvious decreases in euchromatic localization of CTCF, indicating that non-nucleolar CTCF either does not require the poly-ADP-ribosylation cycle, or the maternally-supplied PARG is sufficient for proper localization of that subset of CTCF in salivary gland nuclei.

In populations of S2 cells treated with interfering RNAs directed at PARP and PARG (Figure 4G) we observed increased R1 expression, and increased R2 expression in the case of interfering RNA directed at PARG (Figure 4H). Disrupting the poly-ADP-ribosylation cycle, then, has the predicted effects of CTCF loss from nucleoli, disrupted nucleolar structure, and loss of *rDNA* silencing.

**Figure 5:**
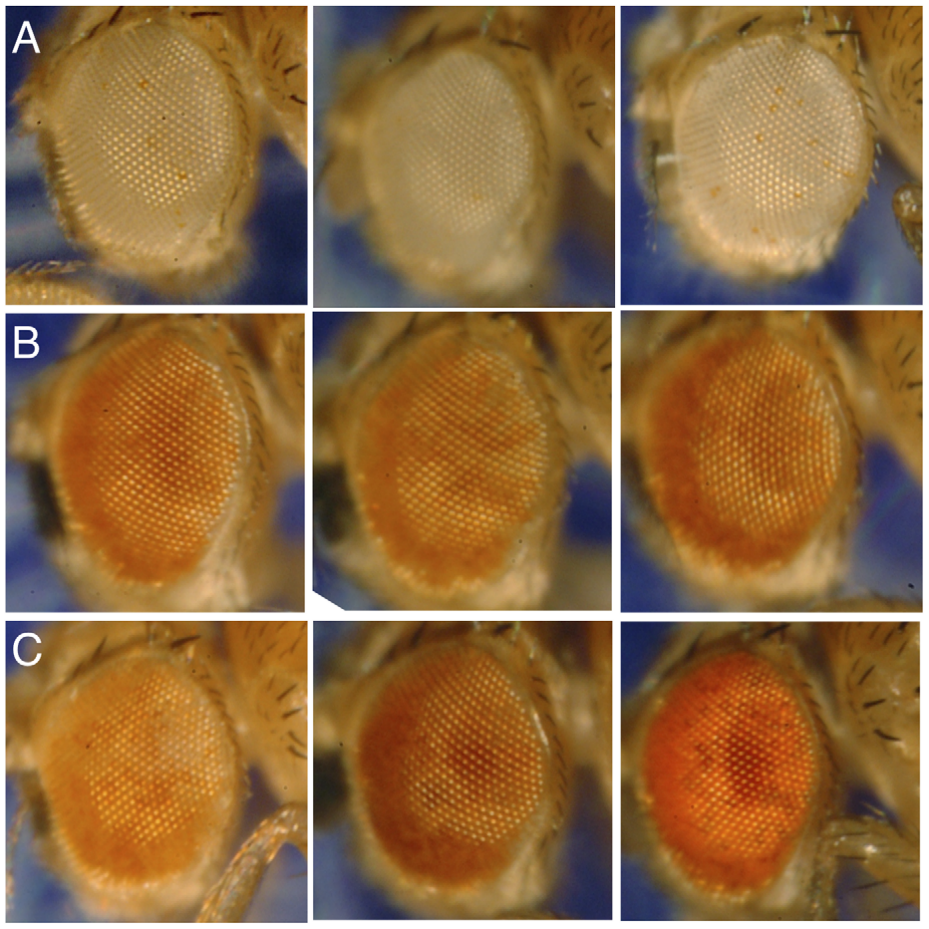
Maternal heterozygosity for *CTCF* suppresses *rDNA*-induced position effect variegation. (A) Expression of *white^+^* from *P*-element *B486* in wild-type flies derived from wild-type (left), *CTCF* ^35.2^/+ (middle), and *Df*(*3L*)*0463*/+ (right) mothers. (B) Expression of *white^+^* from P-element *ROMA* in wild-type flies derived from wild-type (left), *CTCF* ^35.2^/+ (middle), and *Df*(*3L*)*0463*/+ (right) mothers. (C) Expression of *white^+^* from *rDNA*-inserted *P*-element *D285* in wild-type flies derived from wild-type (left), *CTCF* ^35.2^/+ (middle), and *Df*(*3L*)*0463*/+ (right) mothers.

*CTCF* mutation is not known to affect position effect variegation of marker genes near new junctions of centric heterochromatin and euchromatin of inverted chromosomes or transpositions, and we did not observe effects of *CTCF* mutation on either *w* ^m4^ or *w* ^m4h^, two variegating alleles of *white^+^* (data not shown). This is not surprising, since CTCF is not seen to bind to interphase or condensed heterochromatin of *Drosophila* [16]. In addition to the transcriptionally-silent centric heterochromatin, the *rDNA* also induces position effect variegation, as has been detailed in yeast, plants, and *Drosophila* [39], [44], [45]. We reasoned that if CTCF is involved in regulation of the *rDNA*, then mutations in *CTCF* should act as modifier of variegation, but only if that variegation is induced by the *rDNA*. The Karpen laboratory generated a series of *P*-elements inserted in the *Y* chromosome which variegate for both *white* ^+^ and *yellow* ^+^, one of which mapped to the *Y*-linked *rDNA* (line *D285*) [46]. We crossed males carrying this *rDNA*-linked *P*-element to females who were heterozygotes for either a *CTCF* mutation (*CTCF* ^35.2^) or an unrelated chromosomal deficiency that removed *CTCF* (*Df*(*3L*)*0463*). In parallel, females bearing these CTCF alleles were crosses to non-*rDNA* inserts. We compared expression of the *white^+^* marker gene to genetically identical flies who did not have reduced maternal *CTCF* expression. We confirmed that non-*rDNA* variegating *Y*-linked alleles of *white^+^* (Fig. 5A, B) were not affected by maternal heterozygosity of *CTCF*. However, variegation of the *rDNA*-linked transpositional insertion was strongly suppressed by maternal heterozygosity of *CTCF* (Figure 5C), indicating that *CTCF* acts to repress both the RNA Polymerase I-derived *rDNA* and these RNA Polymerase II reporter genes early in development.

## Discussion

We have described the localization of the boundary element protein CCCTC-binding Factor (CTCF) in the nucleolus of *Drosophila*. We find it in cell types taken from different stages of development, and identify a specific binding site in the resident R1 transposable element using chromatin immunoprecipitation. Knockdown of CTCF activity using RNA interference or mutation resulted in disruption of nucleolar structure and derepression of the two *rDNA*-resident transposable elements (R1 and R2), a small increase in steady-state processed rRNA, and an increase in preprocessed rRNAs (indicating increased nascent transcription). Reduction of gene activity of either PARP or PARG, thought necessary for CTCF function, resulted in similar phenotypes. As we predicted, mutation of *CTCF* acted as a suppressor of variegation specifically for an *rDNA* inserted marker gene. Our work extends our understanding of the repertoire of functions for the CTCF protein by demonstrating occupancy, binding, and regulation of *rDNA* by CTCF, and consequence of loss-of-function mutation on *rDNA* behavior.

Our data support a model for *CTCF*-mediated regulation of the *rDNA* consistent with CTCF acting as a direct transcriptional repressor. It is possible that the ability of CTCF to work over long distances, create chromosome interactions that can span hundreds of kilobases, and block both enhancer-promoter interactions and heterochromatin spreading, may contribute to the unique epigenetic regulation demanded by this repeated gene array, which has functional rRNA genes interspersed with silent transposon-inserted or uninterrupted copies. This juxtaposition leads to peculiar behaviors, such as induction of position effect variegation, epigenetic silencing, and nucleolar dominance.

In flies with reduced *CTCF* activity, we observed effects on R1, R2, and uninserted rRNA expression, despite finding binding only in the R1 element. These experimental results are consistent with genetic and cytological data from other laboratories. Eickbush and colleagues have proposed a hypothesis to explain chromosome-specific *rDNA* expression. In their model, clustering of R1 (and R2) elements affects expression of closely-linked cistrons [11], [32]. Arrays with homogeneously interspersed R1 and R2 elements are therefore poised to be inactivated, while arrays with R1 and R2 elements near the array flanks are active and behave as dominant arrays in conditions which elicit nucleolar dominance. Our finding of a sole CTCF binding site in the R1 element might influence uninserted cistrons (or R2-containing cistrons) by nature of their proximity to CTCF-containing R1-inserted cistrons. This “domain” organization of the nucleolar *rDNA* is supported cytologically by the presence of intense discrete chromatin foci which contain R1 and R2 elements [12].

When measuring transcription in cells treated with interfering double-stranded RNAs directed at CTCF, we observed a moderate increase in the *35S* transcription. This effect was not as dramatic as that seen on the R1 and R2 elements, despite those elements being co-linearly transcribed with the rest of the *35S* pre-rRNA. This is consistent with a very long half-life and very high steady-state level of *18S*, *5.8S*/*2S*, and *28S* rRNAs where changes in transcription affect the pool of rRNAs relatively little, and a low abundance of R1 and R2 transcripts where even small changes in transcription are seen as a large “fold” increase in RNA.

Copies of the R1 element exist at the chromocenter, and a truncated R1-like element is found on chromosome *4* [12], [47], [48], [49], and so it is conceivable that our chromatin immunoprecipitation signal is derived from these clusters. However, *Drosophila* CTCF is undetectable at either of these locations ([16] and our data), strongly suggesting that the chIP and Real-Time PCR signals we observe derive from the *rDNA*.

Despite being “unused,” silent copies may serve some purpose in *rDNA* biology and evolution. The mechanism to maintain silent insertions within the arrays may be understood in light of experiments and hypotheses from Peng and Karpen, and work from our laboratory [34], [50], [51]. Normally the *rDNA* arrays are subject to very slow loss or magnification [52], [53], however nucleoli are disrupted and the *rDNA* loss rate increases in animals bearing mutations in chromatin modifying enzymes (the Histone H3-K9 methyltransferase *Su*(*var*)*3*–*9* or the methylhistone binding protein Heterochromatin Protein 1) or those with experimentally shortened arrays [34], [35]. We believe the disruption of nucleolar structure by reduction of *CTCF*, *PARP*, or *PARG* to be similar. In all of these cases, we envision that *rDNA* transcription is increased (by removal of transcriptional repression as in this study, by removal of silencing chromatin modifications in the Peng and Karpen studies, or by compensatory mechanisms to maximize *rDNA* transcription of short arrays in our previous work), resulting in increased intrachromosomal recombination and subsequent nucleolar fragmentation.

Our results demonstrate a clear role for *CTCF* in *rDNA* regulation, but formally we cannot show that this effect is direct, since disruption of *CTCF* or *PARP*/*PARG* are expected to have pleiotropic effects on other genes in the genome. However, our demonstration of CTCF occupancy in the nucleolus by immunofluorescence and chromatin immunoprecipitation both suggest the regulation is direct. This is supported by data from human cells, which shows CTCF to be in the nucleolus by immunofluorescence [20] and chromatin immunoprecipitation [24], and hence we do not believe that *CTCF*-dependent regulation of the *35S* rRNA gene is unique to *Drosophila*, as the presence of consensus CTCF binding sites within the *rDNA* repeat unit is conserved in humans and *Xenopus* [24], [31], conserved interactions have been shown between *Myc*, *cohesin*, and *CTCF*, all of which impact rRNA regulation [54], [55], [56], and overexpression of CTCF truncations affects nucleolar transcription in mammals [20]. Many proteins are facultative or transient members of the nucleolar protein fraction, and dynamic membership of CTCF might be a general mode of rRNA gene regulation.

## Materials and Methods

### Fly Husbandry and stocks

Fly stocks were maintained at 25°C on standard cornmeal-based medium, supplemented with yeast. Stock *w* ^1118^ *Parg* ^27.1^/*FM7i*, *P*{*w* ^+mC^ = *ActGFP*}*JMR3* was obtained from the Kyoto Stock Center. *CTCF* ^35.2^/*TM6B*, *Tb* and Df(3L)0463/TM6B, Tb were kindly provided by Dr. Pamela Geyer at the University of Iowa and was characterized in a previous study [16]. *D285*, *B486* and *ROMA* are stocks containing *P*{*SUPorP*} transposons to the *Y* chromosome [44]. *C*(*1*)*DX* is *C*(*1*)*DX*, *y* ^1^ *f* ^1^ *bb* ^0^.

### Immunofuorescence and Confocal Microscopy

Salivary glands were dissected in phosphate-buffered saline (PBS), incubated for 20 seconds in PBS containing 1% Triton X-100 and 3.7% formaldehyde, then transferred to a solution of 3.7% formaldehyde and 50% acetic acid for 2 minutes and immediately squashed. Slides were washed twice in PBS for 10 minutes, transferred to PBS containing 0.1% Triton X-100 for 10 minutes and blocked with PBS containing 1% BSA for one hour at room temperature. Primary antibody was added and slides were incubated overnight at 4°C. Rabbit anti-CTCF antibody was used at 1/500 dilution. Mouse anti-Fibrillarin antibody was purchased from Abcam and was used at 1/200 dilution. Slides were washed twice in PBS containing 1% BSA and incubated with secondary antibody at room temperature for 2 hours. Goat anti-rabbit conjugated to rhodamine and goat anti-mouse conjugated to DL488 (Jackson ImmunoResearch) were each used at 1/200 dilution. Slides were immersed in 4′,6-diamidino-2-phenylindole (DAPI, at 1 ng/mL) for 5 minutes, washed, and mounted in Vectashield (Vector Laboratories).

Brains were dissected in 0.7% (w/v) sodium chloride, incubated for 7 minutes in 0.5% (w/v) Sodium Citrate, transferred to a solution of Methanol/Formaldehyde/water (11/11/2 ratio) for 30 seconds, then transferred and squashed in 45% acetic acid. Thereafter, the slides were treated as above.

For whole mount salivary glands, dissection was performed in PBS containing 1% Triton X-100, then transferred to PBT (PBS containing 0.1% Tween-80) and fixed in PBS containing 1% Triton X-100 and 3.7% formaldehyde. The tissue was blocked for 1 hour in PBT supplemented with 1% BSA. Primary antibodies were diluted in PNBT (PBT containing 1% BSA and 500 mM NaCl) and incubated overnight at 4°C. The tissue was then washed in PNBT and incubated in secondary antibody for 2 hours, then washed and mounted in 70% glycerol.

For confocal microscopy, sequential excitation was performed at 488 nm (for DL488), 543 nm (for Rhodamine) and 405 nm (for DAPI) in an Olympus FV1000 confocal microscope. The images were processed using FV19-ASW 1.7 Viewer.

S2 cell immunofluorescence was performed as described [57]. Quantification of CTCF and fibrillarin immunofluorescence signal was done by independently capturing DAPI (for DNA), and rhodamine and fluorescein (for protein epitopes) channels and exporting to NIH Image-J. Entire fluorescence signals were integrated and divided by exposure time to determine intensity/time in arbitrary units. Individual nucleus measurements were normalized to DAPI signals to create datasets amenable to graphical and statistical comparison.

### Chromatin Immunoprecipitation

ChIP experiments were carried out as described [58], with some modifications. Briefly, 200 µL of third instar larvae were used per immunoprecipitation reaction; chromatin was cross-linked for 10 minutes at room temperature with 1% formaldehyde. Sonication was performed for 8 minutes, with 20 second pulses followed by 40 seconds “cooling off” period. After confirming fragment size averaging approximately 500 base pairs, protein concentration was estimated using the Bradford assay. 500 µg of chromatin was incubated with 3-4 µL of rabbit anti-CTCF antibody. 50 µg of chromatin was set apart as input. For all buffers, PMSF and Complete Protease Inhibitor Cocktail tablets (Roche) were used as protease inhibitors. DNA was diluted in 1/20 for antibody and no antibody samples and 1/300 for input. Real Time PCR was used for quantification of precipitated DNA.

Primers used to amplify regions shown in Figure 2 are: 1 GGTTGCCAAACAGCTCGTCATC and CGAGGTGTTTGGCTACTCTTG, 2 GCCAAACACCTCGTCATCAA and GAGAGGTCGGCAACCAC, 3 GAGTAGCCAAACACCTCGTC and GAGAGGTCGGCAACCAC, 4 GCTGTTCTACGACAGAGGGTTC and CAATATGAGAGGTCGGCAACCAC, 5 GGTAGGCAGTGGTTGCCG and GGAGCCAAGTCCCGTGTTC, 6 ATTACCTGCCTGTAAAGTTGG and CCGAGCGCACATGATAATTCTTCC, 7 TTCTGGTTGATCCTGCCAGTAG and CGTGTGTACTTAGACATGCATGGC, 8 AGCCTGAGAAACGGCTACCA and AGCTGGGAGTGGGTAATTTACG, 9 GTAAGCGTATTACCGGTGGAGTTC and GTACCGGCCCACAATAACACTCG, 10 CGACGCGAGAGGTGAAATTC and TATCTGATCGCCTTCGAACCTC, 11 CGTACCTGTTGGTTTGTCCCAT and TACTTTCATTGTAGCGCGCGTGC, 12 GCATTGATTACGTCCCTGCCC and CCG TAACACGCAAGGCG, 13 GTTAGTGTGGGGCTTGGC and CGC CGTTGTTGTAAGTACTCG, 14 GTTGTACCTGGCATCCATCAGG and CTGGTTGGTTATGGGGTTTGC, 15 GAAACTAAGACATTTCGCAAC and CACCATTTTACTGGCATATATCAATTCC, 16 CTGTGCGTCATCGTGTGAACT and GTACATAACAGCATGGACTGCG, 17 CCTCAACTCATATGGGACTACCC and CGCTCCATACACTGCATCTCAC, 18 GTGAGATGCAGTGTATGGAGCG and GCTGCACTATCAAGCAACACG, 19 GATTCAGGATACCTTCGGGACC and GAACGCCCCGGGATTGTG, 20 GAGTATAGGGGCGAAAGACCAA and GCACCAGCTATCCTGAGGG, 21 GGAGTGTGTAACAACTCACCTGC and GGTATACAACTTAAGCGCCATCC, 22 CTAAGTTCAAGGCGAAAGCCG and CGGATACTCAACAGGTTACGG, 23 GCAGCTGGTCTCCAAGGTG and CCCAGAACGAGCACATAAACC, 24 CAAGTAAGCGCGGGTCAACGG and CCCTTGGCTGTGGTTTCGCTAG, 25 CGGGCTTGGAATAATTAGCGG and CCGAGGTGTAATATCTCCCAC, 26 GGACATTGCCAGGTAGGGAG and GCTGTCCCTGTGTGTACTGAAC, 27 CCGTGCTGGACTGCAATG and CATTGGCATCACATCCATTGTCG, 28 GGGACAGCTTAGTGCACTCTAC and CCAGCAATCGTATGCTCGCTG, 29 GCGGAAGCAGTGCCTC and CAGTTTCGCCTGCGTTGG, 30 CGCTTCGTGGGAGATCATGC and CCCAATCTCCGTGCACTTC, 31 CCCCGGAAGTTGCTAATCTAACC and GGGAGTGATGGAGTTGTTTCCG. Primers with sequence AAGTTGTGGACGAGGCCAAC and CGGTTCTCGTCCGATCACCGA were used as endogenous control which amplified a fragment of the *5S rDNA*.

### Reverse Transcriptase Real Time PCR (RT-Q PCR)

RNA from adult flies or S2 cells were extracted as described [59]. Primers used for the reverse transcriptase reaction were: 35S GTACCGGCCCACAATAACACTCG, R1 CCAGCAATCGTATGCTCGCTG, R2 GCCAACACTGTGTGTGGTCA, uninserted R1 and R2 CCGAGGTGTAATATCTCCCAC *Rho1* CTTAGCCGAACACTCCAAATAGG. Real-Time PCR: 35S AGCCTGAGAAACGGCTACCA and AGCTGGGAGTGGGTAATTTACG, R1 (b) GCCTCGTCATCTAATTAGTGACGCGC and (c) CCACGAGCGCAACGAAAACACG, R2 (e) GGATGTGATGCTCCCGAAAC and (f) CAAGTCCCCGCTTGATTCGA, uninserted R1 and R2 (a) GCCTCGTCATCTAATTAGTGACGCGC and (d) CCCTTGGCTGTGGTTTCGCTAG, *Rho1* GTGGAGCTGGCCTTGTGGG and CTAGCGAATCGGGTGAATCCACTG, *2S*-ITS2 junction GGACTACATATGGTTGAGGGTTG and GCTAGACATTTCTCAGTATTATTTG, ITS2-*28S* junction GAATTGTCTCTTATTAATGATTCGG and GTAGTCCCATATGAGTTGAGG. Reverse Transcriptase reaction product cDNA was diluted 1∶25 to 1∶60 as determined empirically with test samples to optimize melting curve (single-peak) and crossing threshold (not greater than cycle 29).

The primers used to quantify *CTCF*, *PARP* and *PARG* mRNA levels were: *CTCF* ACGAGGAGGTGTTGGTCAAG and ATCATCGTCGTCCTCGAAA, *PARP* GTTTGCAGAAGAGCTCGGAATTC and either CCCCAACTACAAATACATGTGC or GCTGAACTTTGTAGTAGGAGTTC, *PARG* CGCCGCAGAGCAAGTGC and either CTTCGACATCCTGGCGCAG or GGCGTTCTTGTGGTGCTTG.

### Genomic DNA extraction

Females of genotype *C*(*1*)*DX*, *y* ^1^ *f* ^1^ *bb* ^0^ were crossed to *y* ^1^ *w* ^1118^/*Y*; *CTCF*/*TM6B*, *Tb* and genomic DNA from Tubby and non-Tubby males was extracted and subjected to Real-Time PCR [38].

### RNAi in S2 cells

*Drosophila* Schneider 2 (S2) cells were culture in Schneider media supplemented with 50 µg/mL streptomycin, 50 µg/mL penicillin and 10% heat-inactivated fetal bovine serum (GIBCO). After reaching a density of 10^6^ cells/mL, they were washed twice in serum free medium. 15 µg of double-stranded RNA was added to 1 mL of S2 cells resuspended in serum free medium, mixed by swirling and incubated at room temperature for 1 hour. 2 mL of medium containing serum was then added, and cells were cultured at 25°C for three days. An aliquot was taken on every day for five days and samples were analyzed by immunofluorescence and RT-Q PCR. Double-stranded RNA was generated by PCR amplifying gene sequence using primers that contained the T7 RNA Polymerase promoter: *CTCF* ACTAAAGGCCCACAAGCTCA and TGACAGTGCCATCTTTCTGC, *PARP* GAGTTCGACACGAGCGAGT and GCGCCTTGCTTCTCCTT, *PARG* CCGGCAGTTCTGGAGAA and CCATGAGATCCTCGCGATATT, *LacZ* *TAATACGACTCACTATAGGAGGTATTCGCTG and TAATACGACTCACTATAGGCGATCGTAATCACC. RNA was transcribed using the T7 MEGAscript Kit (Ambion) without deviation from the manufacturer's instructions.*

## References

[1] McStay B, Grummt I (2008). The epigenetics of rRNA genes: from molecular to chromosome biology.. Annu Rev Cell Dev Biol.

[2] Miller OL, Beatty BR (1969). Visualization of nucleolar genes.. Science.

[3] Percipalle P, Fomproix N, Cavellan E, Voit R, Reimer G (2006). The chromatin remodelling complex WSTF-SNF2h interacts with nuclear myosin 1 and has a role in RNA polymerase I transcription.. EMBO Rep.

[4] Langst G, Becker PB, Grummt I (1998). TTF-I determines the chromatin architecture of the active rDNA promoter.. EMBO J.

[5] Huang J, Moazed D (2003). Association of the RENT complex with nontranscribed and coding regions of rDNA and a regional requirement for the replication fork block protein Fob1 in rDNA silencing.. Genes Dev.

[6] Conconi A, Widmer RM, Koller T, Sogo JM (1989). Two different chromatin structures coexist in ribosomal RNA genes throughout the cell cycle.. Cell.

[7] Noma K, Cam HP, Maraia RJ, Grewal SI (2006). A role for TFIIIC transcription factor complex in genome organization.. Cell.

[8] Donze D, Adams CR, Rine J, Kamakaka RT (1999). The boundaries of the silenced HMR domain in Saccharomyces cerevisiae.. Genes Dev.

[9] Jakubczak JL, Zenni MK, Woodruff RC, Eickbush TH (1992). Turnover of R1 (type I) and R2 (type II) retrotransposable elements in the ribosomal DNA of Drosophila melanogaster.. Genetics.

[10] Jamrich M, Miller OL (1984). The rare transcripts of interrupted rRNA genes in Drosophila melanogaster are processed or degraded during synthesis.. EMBO J.

[11] Eickbush DG, Ye J, Zhang X, Burke WD, Eickbush TH (2008). Epigenetic regulation of retrotransposons within the nucleolus of Drosophila.. Mol Cell Biol.

[12] Plata MP, Kang HJ, Zhang S, Kuruganti S, Hsu SJ (2008). Changes in chromatin structure correlate with transcriptional activity of nucleolar rDNA in polytene chromosomes.. Chromosoma.

[13] Eickbush DG, Eickbush TH (2010). R2 retrotransposons encode a self-cleaving ribozyme for processing from an rRNA cotranscript.. Mol Cell Biol.

[14] Ohlsson R, Bartkuhn M, Renkawitz R (2010). CTCF shapes chromatin by multiple mechanisms: the impact of 20 years of CTCF research on understanding the workings of chromatin.. Chromosoma.

[15] Moon H, Filippova G, Loukinov D, Pugacheva E, Chen Q (2005). CTCF is conserved from Drosophila to humans and confers enhancer blocking of the Fab-8 insulator.. EMBO Rep.

[16] Mohan M, Bartkuhn M, Herold M, Philippen A, Heinl N (2007). The Drosophila insulator proteins CTCF and CP190 link enhancer blocking to body patterning.. EMBO J.

[17] Kurukuti S, Tiwari VK, Tavoosidana G, Pugacheva E, Murrell A (2006). CTCF binding at the H19 imprinting control region mediates maternally inherited higher-order chromatin conformation to restrict enhancer access to Igf2.. Proc Natl Acad Sci U S A.

[18] Donohoe ME, Zhang LF, Xu N, Shi Y, Lee JT (2007). Identification of a Ctcf cofactor, Yy1, for the X chromosome binary switch.. Mol Cell.

[19] Holohan EE, Kwong C, Adryan B, Bartkuhn M, Herold M (2007). CTCF genomic binding sites in Drosophila and the organisation of the bithorax complex.. PLoS Genet.

[20] Torrano V, Navascues J, Docquier F, Zhang R, Burke LJ (2006). Targeting of CTCF to the nucleolus inhibits nucleolar transcription through a poly(ADP-ribosyl)ation-dependent mechanism.. J Cell Sci.

[21] Yusufzai TM, Tagami H, Nakatani Y, Felsenfeld G (2004). CTCF tethers an insulator to subnuclear sites, suggesting shared insulator mechanisms across species.. Mol Cell.

[22] Bernardi R, Scaglioni PP, Bergmann S, Horn HF, Vousden KH (2004). PML regulates p53 stability by sequestering Mdm2 to the nucleolus.. Nat Cell Biol.

[23] Weber JD, Taylor LJ, Roussel MF, Sherr CJ, Bar-Sagi D (1999). Nucleolar Arf sequesters Mdm2 and activates p53.. Nat Cell Biol.

[24] van de Nobelen S, Rosa-Garrido M, Leers J, Heath H, Soochit W (2010). CTCF regulates the local epigenetic state of ribosomal DNA repeats.. Epigenetics Chromatin.

[25] Gerasimova TI, Corces VG (1998). Polycomb and trithorax group proteins mediate the function of a chromatin insulator.. Cell.

[26] Gerasimova TI, Gdula DA, Gerasimov DV, Simonova O, Corces VG (1995). A Drosophila protein that imparts directionality on a chromatin insulator is an enhancer of position-effect variegation.. Cell.

[27] Smith ST, Wickramasinghe P, Olson A, Loukinov D, Lin L (2009). Genome wide ChIP-chip analyses reveal important roles for CTCF in Drosophila genome organization.. Dev Biol.

[28] Burcin M, Arnold R, Lutz M, Kaiser B, Runge D (1997). Negative protein 1, which is required for function of the chicken lysozyme gene silencer in conjunction with hormone receptors, is identical to the multivalent zinc finger repressor CTCF.. Mol Cell Biol.

[29] Stage DE, Eickbush TH (2007). Sequence variation within the rRNA gene loci of 12 Drosophila species.. Genome Res.

[30] Bao L, Zhou M, Cui Y (2008). CTCFBSDB: a CTCF-binding site database for characterization of vertebrate genomic insulators.. Nucleic Acids Res.

[31] Nobelen Svd (2008). Touched by CTCF: Analysis of a Multi-Functional Zinc Finger Protein.. Rotterdam: Erasmus.

[32] Zhou J, Eickbush TH (2009). The pattern of R2 retrotransposon activity in natural populations of Drosophila simulans reflects the dynamic nature of the rDNA locus.. PLoS Genet.

[33] Winkles JA, Phillips WH, Grainger RM (1985). Drosophila ribosomal RNA stability increases during slow growth conditions.. J Biol Chem.

[34] Paredes S, Maggert KA (2009). Ribosomal DNA contributes to global chromatin regulation.. Proc Natl Acad Sci U S A.

[35] Peng JC, Karpen GH (2007). H3K9 methylation and RNA interference regulate nucleolar organization and repeated DNA stability.. Nat Cell Biol.

[36] Perrin L, Demakova O, Fanti L, Kallenbach S, Saingery S (1998). Dynamics of the sub-nuclear distribution of Modulo and the regulation of position-effect variegation by nucleolus in Drosophila.. J Cell Sci.

[37] Gottlieb S, Esposito RE (1989). A new role for a yeast transcriptional silencer gene, SIR2, in regulation of recombination in ribosomal DNA.. Cell.

[38] Paredes S, Maggert KA (2009). Expression of I-CreI Endonuclease Generates Deletions Within the rDNA of Drosophila.. Genetics.

[39] Ashburner M, Golic KG, Hawley RS (2005). Drosophila : a laboratory handbook..

[40] Yu W, Ginjala V, Pant V, Chernukhin I, Whitehead J (2004). Poly(ADP-ribosyl)ation regulates CTCF-dependent chromatin insulation.. Nat Genet.

[41] Tulin A, Spradling A (2003). Chromatin loosening by poly(ADP)-ribose polymerase (PARP) at Drosophila puff loci.. Science.

[42] Tulin A, Naumova NM, Menon AK, Spradling AC (2006). Drosophila poly(ADP-ribose) glycohydrolase mediates chromatin structure and SIR2-dependent silencing.. Genetics.

[43] Tulin A, Stewart D, Spradling AC (2002). The Drosophila heterochromatic gene encoding poly(ADP-ribose) polymerase (PARP) is required to modulate chromatin structure during development.. Genes Dev.

[44] Maggert KA, Golic KG (2002). The Y chromosome of Drosophila melanogaster exhibits chromosome-wide imprinting.. Genetics.

[45] Fritze CE, Verschueren K, Strich R, Easton Esposito R (1997). Direct evidence for SIR2 modulation of chromatin structure in yeast rDNA.. EMBO J.

[46] Konev AY, Yan CM, Acevedo D, Kennedy C, Ward E (2003). Genetics of P-element transposition into Drosophila melanogaster centric heterochromatin.. Genetics.

[47] Kidd SJ, Glover DM (1980). A DNA segment from D. melanogaster which contains five tandemly repeating units homologous to the major rDNA insertion.. Cell.

[48] Browne MJ, Read CA, Roiha H, Glover DM (1984). Site specific insertion of a type I rDNA element into a unique sequence in the Drosophila melanogaster genome.. Nucleic Acids Res.

[49] Steffensen DM, Appels R, Peacock WJ (1981). The distribution of two highly repeated DNA sequences within Drosophila melanogaster chromosomes.. Chromosoma.

[50] Peng JC, Karpen GH (2009). Heterochromatic genome stability requires regulators of histone H3 K9 methylation.. PLoS Genet.

[51] Peng JC, Karpen GH (2008). Epigenetic regulation of heterochromatic DNA stability.. Curr Opin Genet Dev.

[52] Cohen S, Yacobi K, Segal D (2003). Extrachromosomal circular DNA of tandemly repeated genomic sequences in Drosophila.. Genome Res.

[53] Hawley RS, Tartof KD (1985). A two-stage model for the control of rDNA magnification.. Genetics.

[54] Rubio ED, Reiss DJ, Welcsh PL, Disteche CM, Filippova GN (2008). CTCF physically links cohesin to chromatin.. Proc Natl Acad Sci U S A.

[55] Filippova GN, Fagerlie S, Klenova EM, Myers C, Dehner Y (1996). An exceptionally conserved transcriptional repressor, CTCF, employs different combinations of zinc fingers to bind diverged promoter sequences of avian and mammalian c-myc oncogenes.. Mol Cell Biol.

[56] Huang J, Brito IL, Villen J, Gygi SP, Amon A (2006). Inhibition of homologous recombination by a cohesin-associated clamp complex recruited to the rDNA recombination enhancer.. Genes Dev.

[57] Angshuman S, Cordula S (2007). An Approach for Immunofluorescence of Drosophila S2 Cells.. Cold Spring Harb Protoc.

[58] Yu W, Zheng H, Houl JH, Dauwalder B, Hardin PE (2006). PER-dependent rhythms in CLK phosphorylation and E-box binding regulate circadian transcription.. Genes Dev.

[59] Bogart K, Andrews J (2006). Extraction of Total RNA from Drosophila The Center for Genomics and Bioinformatics, Indiana University, Bloomington, Indiana.

